# Pathological features of the differentiation landscape in esophageal squamous cell cancer and their correlations with prognosis

**DOI:** 10.3389/fonc.2024.1442212

**Published:** 2024-12-06

**Authors:** Jiaying Deng, Lei Zhang, Zezhou Wang, Bin Li, Jiaqing Xiang, Longfei Ma, Hongcheng Zhu, Yuan Li, Kuaile Zhao

**Affiliations:** ^1^ Department of Radiation Oncology, Fudan University Shanghai Cancer Center, Shanghai, China; ^2^ Department of Oncology, Shanghai Medical College, Fudan University, Shanghai, China; ^3^ Shanghai Clinical Research Center for Radiation Oncology, Shanghai, China; ^4^ Shanghai Key Laboratory of Radiation Oncology, Shanghai, China; ^5^ Department of Pathology, Zhongshan Hospital, Fudan University, Shanghai, China; ^6^ Department of Cancer Prevention, Fudan University Shanghai Cancer Center, Shanghai, China; ^7^ Department of Thoracic Surgery and State Key Laboratory of Genetic Engineering, Fudan University Shanghai Cancer Center, Shanghai, China; ^8^ Institute of Thoracic Oncology, Fudan University, Shanghai, China; ^9^ Department of Pathology, Fudan University Shanghai Cancer Center, Shanghai, China

**Keywords:** differentiation, pathological features, esophageal squamous cell carcinoma, nuclear diameter, prognosis

## Abstract

**Background:**

For esophageal squamous cell carcinoma (ESCC), universally accepted pathological criteria for classification by differentiation degree are lacking. Tumor budding, single-cell invasion, and nuclear grade, recognized as prognostic factors in other carcinomas, have rarely been investigated for their correlation with differentiation and prognosis in ESCC. This study aims to determine if pathological findings can predict differentiation degree and prognosis in ESCC.

**Patients and methods:**

This study reviewed tumor slides from 326 patients who underwent surgery for ESCC between 2007 and 2012. Tumors were evaluated for subtypes, tumor nest size, tumor stroma, and nuclear grade (nuclear diameter and mitosis) across different differentiation groups. Overall survival (OS) and disease-free survival (DFS) were estimated using the Kaplan-Meier method, with group differences assessed using the stratified log-rank test and Cox proportional hazards model.

**Results:**

The mean values of tumor budding invasion margins in well, moderately, and poorly differentiated groups were 25.3%, 30.7%, and 36.3%, respectively. Mean tumor budding/10HPFs were 8.0, 10.3, and 13.0, respectively. Well-differentiated tumors showed more keratinizing subtypes, smaller tumor budding invasion margins, more Grade 1 tumor budding (0-4 cells), absence of single-cell invasion, larger nuclear diameter (≥5 lymphocytes), higher mitotic counts, more submucosal invasion, and less lymphovascular invasion. Conversely, poorly differentiated tumors exhibited opposite characteristics. Multivariate analyses identified the nuclear diameter as independent prognostic factors for OS and DFS.

**Conclusions:**

Pathological features can stratify the differentiation landscape in ESCC patients. The nuclear diameter (4 lymphocytes) can help predict prognosis in ESCC than other pathological features.

**Implications for practice:**

We first time report the mean values of tumor budding invasion margins and tumor budding/10HPF in well, moderately, and poorly differentiated groups for esophageal squamous cell carcinoma. The landscape of well differentiation was depicted with more keratinizing subtypes, smaller tumor budding invasion margins, more Grade 1 tumor budding (0-4 cells), absence of single-cell invasion, larger nuclear diameter (≥5 lymphocytes), higher mitotic counts, and less lymphovascular invasion. The nuclear diameter as independent prognostic factors for prognosis. The findings indicate that pathological features can stratify the differentiation landscape in ESCC patients and offer novel insight into definition of well or moderately differentiation.

## Background

No universally accepted histological classification exists for esophageal squamous cell carcinoma (ESCC), leading to the adaptation of lung squamous cell carcinoma (LSCC) grading systems based on keratinization levels for ESCC. The distinction of well and moderate differentiation is not so clear thus hard to define the absolute well or moderate differentiation. Comprehensive pathological analyses in LSCC have led to a proposed grading system that aids prognosis prediction (1, 2). However, an equivalent system for ESCC that predicts clinical outcomes has yet to be established.

Tumor budding, identified by isolated small tumor nests of fewer than five cells at the invasive tumor edge, is a known prognostic indicator initially for colorectal cancer (3, 4). Single-cell invasion, a subtype of tumor budding, correlates with reduced overall survival in LSCC (1). In contrast, in ESCC, tumor budding relates to lymph node metastasis and the effectiveness of neoadjuvant chemotherapy but not single-cell invasion (5, 6). Moreover, the size of tumor nests, with minimal tumor nest (MTN) size at the invasive tumor front (ITF), is considered a poor prognostic factor in head and neck squamous cell carcinomas (7). MTN can be subclassified according to the cell number, that is the single cell invasion and nest tumor cells including large nest (≥15 tumor cells), intermediate nest (5–14 tumor cells), small nest (2–4 tumor cells) (1, 8). The ITF, different biologically from the tumor center, often exhibits epithelial-mesenchymal transition (EMT), enhancing migration and invasion capabilities (9–11). Research on ITF in ESCC remains limited.

In terms of nuclear grading, such as mitotic count and nuclear diameter has been established in breast cancer and lung adenocarcinoma (12, 13). A higher mitotic count is an independent predictor of recurrence in lung adenocarcinoma contrasting with LSCC (14). Large nuclear diameters defined as greater than four small lymphocytes have been associated with worse overall survival in LSCC (1). Yet, a rigorous investigation into a grading system for ESCC that predicts clinical outcomes is still lacking. We all know that tumor progression is intricately linked with its microenvironment. The tumor-stroma ratio (TSR), the proportion of tumor cells to stromal cells within a tumor, is a prognostic indicator in several types of cancer, including colon, breast, cervical, and non-small cell lung cancer. A threshold to differentiate stroma-poor and stroma-rich always is 50%, with a stroma-rich environment associated with a poorer prognosis—a trend consistent across the cancers mentioned above (15–17). Yet, the relationship between TSR, tumor differentiation, and the clinical relevance of TSR in ESCC remains unexplored.

This study, involving a significant number of patients with surgically resected ESCC, undertook a detailed analysis of pathological factors, including histologic subtype, tumor budding, tumor nest size, and nuclear grade, to evaluate their variations across different degrees of differentiation. We also aimed to determine whether any of these pathological factors could predict clinical outcomes—overall survival (OS) and disease-free survival (DFS)—independently of the pathological stage.

## Methods

### Patients

This retrospective study received the endorsement of the Institutional Review Board at Fudan University Shanghai Cancer Center (FUSCC). Our comprehensive review encompassed all patients diagnosed with ESCC who were subjected to surgical resection from 2007 to 2012. Through diligent examination, histologic evaluation was deemed feasible for 326 patients, courtesy of the accessibility to their tumor slides. The classification of disease stages was meticulously determined, adhering to the the 7th edition of TNM Staging.

### Histologic evaluation and criterion

All available H&E-stained slides underwent a thorough review by the same experienced pathologist, Dr. L.Z., employing an Olympus BX51 microscope (Olympus, Tokyo, Japan) with a standard 22-mm diameter eyepiece. Tumors were graded by the degree of differentiation into well, moderately, and poorly differentiated, in accordance with the 2004 WHO classification of lung carcinomas. In the well-differentiation criterion, tumor nests displayed prominent keratinization both in layered formations and within the cytoplasm, alongside noticeable intercellular bridges, hallmarking their well-differentiated status. Conversely, poorly differentiated tumors were noted for presenting squamous structures only in limited regions, indicating a significant deviation from normal cell differentiation. Moderately differentiated tumors demonstrated an intermediate level of differentiation that lies between well and poorly differentiated tumors.

**Figure 1:**
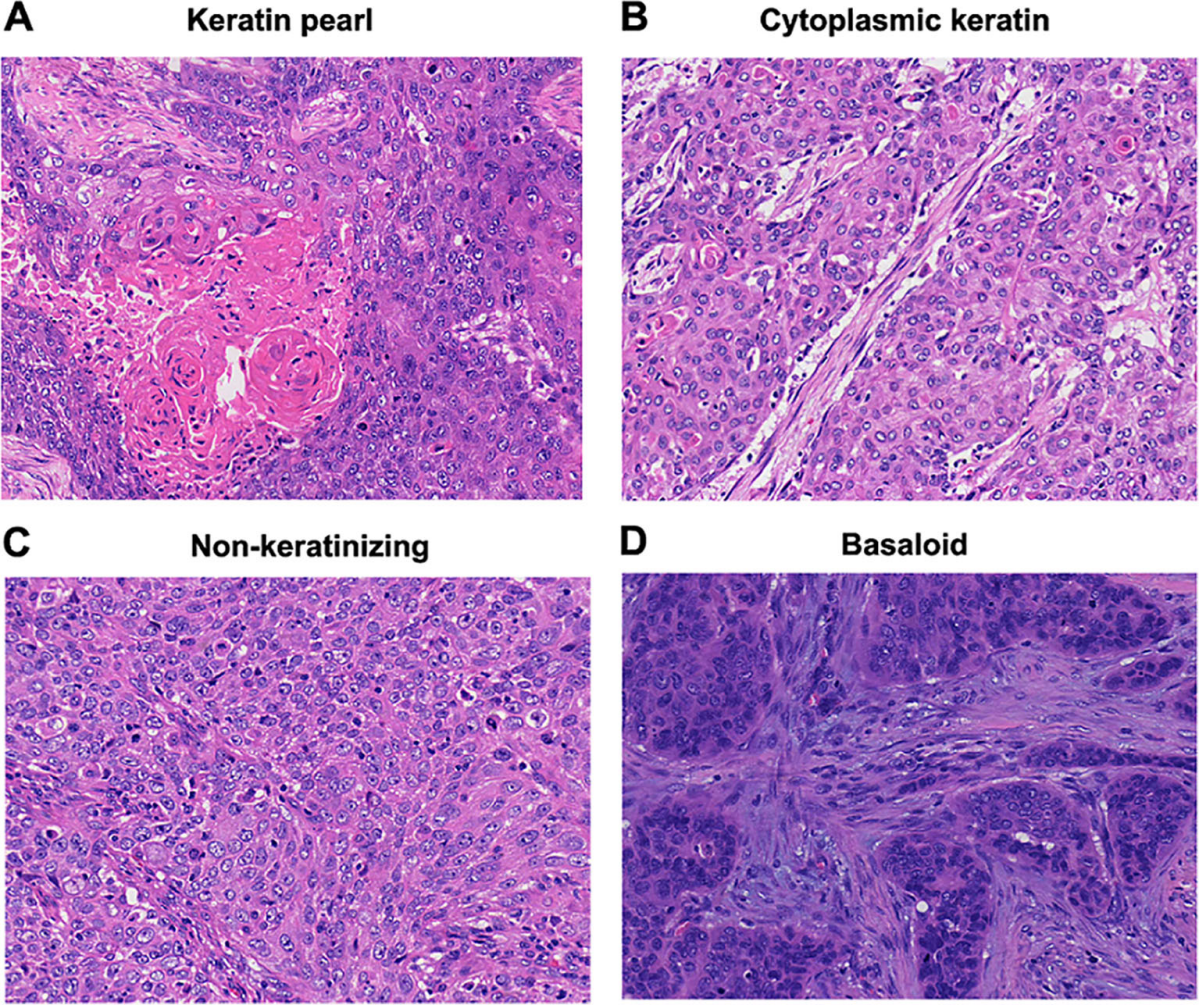
Histologic subtypes (hematoxylin and eosinstain; original magnification, x200: **(A–D)**. **(A)** keratinizing subtype with layered keratin. **(B)** Keratinizing subtype with cytoplasmic keratinization. **(C)** Nonkeratinizing subtype. **(D)** Basaloid subtype.

Histologic subtyping of ESCC was classified according to the 2005 WHO Classification in nasopharyngeal carcinomas; that identified tumors as nonkeratinizing, keratinizing, and basaloid squamous cell carcinomas (18). The percentage of keratinizing pattern, including layered (**Figure 1A**) and cytoplasmic keratinization (**Figure 1B**), was recorded. The keratinizing subtype was defined as greater than or equal to 5% keratinizing pattern of the entire tumor whereas nonkeratinizing subtypes were defined as having less than 5% keratinizing pattern (**Figure 1C**). The basaloid subtype was distinctly identified by its pronounced peripheral palisading of tumor cells, scant cytoplasm and an elevated presence of hyperchromatic nuclei (**Figure 1D**). The basaloid subtype was defined as the percentage of basaloid pattern greater than 50% as previously recommended (19).

**Figure 2:**
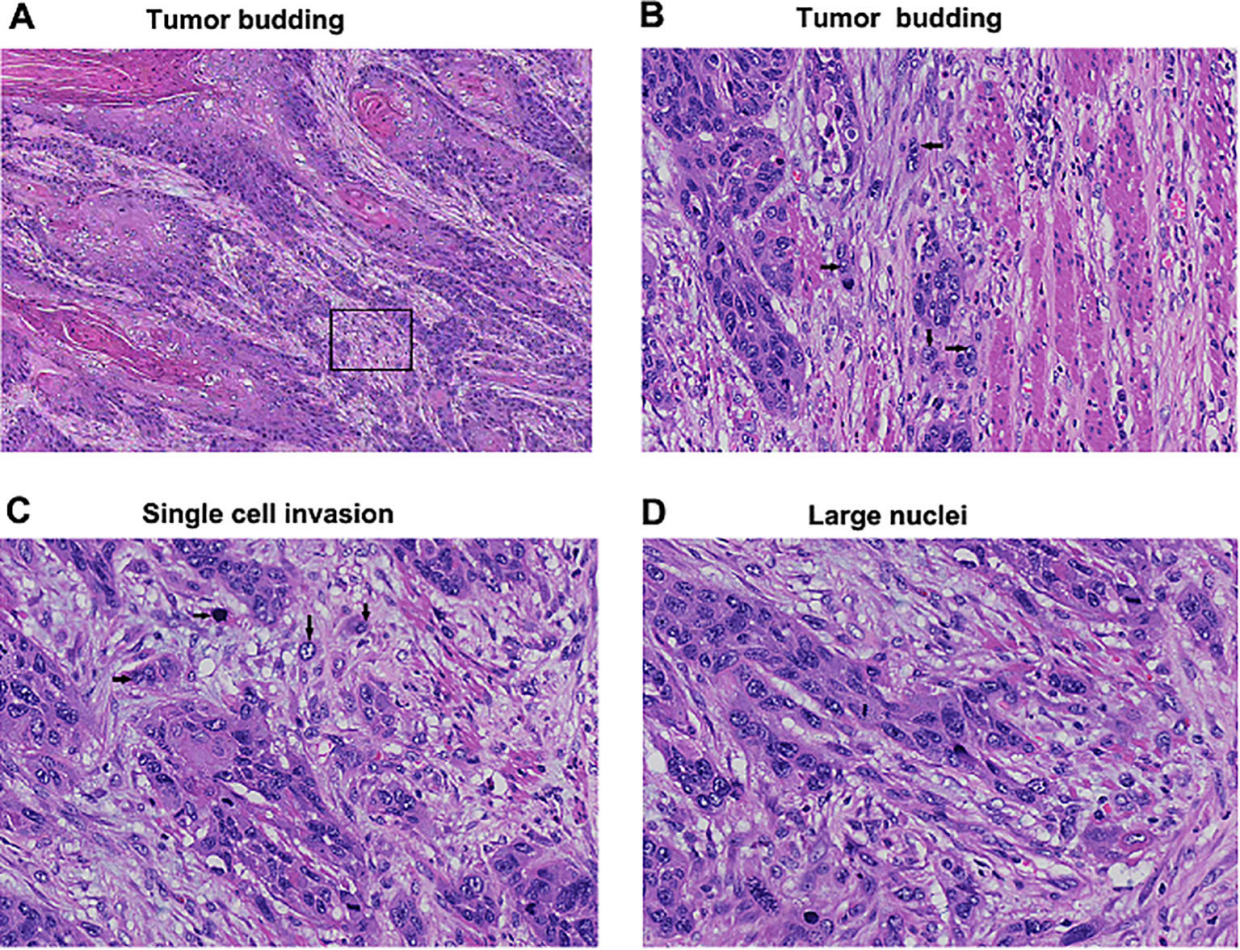
Tumor budding and single cell invasion (hematoxylin and eosin-stain; original magnification, ×40: A, ×400: **(C, D)**. **(A)** Tumor budding identified in invasive tumor edge. **(B)** Higher magnification of a square box showing tumor budding composed of less than five tumor cells (arrows). **(C)** Single cell invasion of tumor cells in stroma (arrows). **(D)** Large nuclei defined as greater than four small lymphocytes in diameter.

Upon conducting a thorough examination of the entire series of tumor slides under intermediate-power magnification at ×100, tumor budding and minimal cell nest were assessed within the regions demonstrating the highest degree of invasiveness. Tumor budding was defined as small tumor nests composed of less than five tumor cells (**Figures 2A, B**), and they were counted both under 1 and 10 high-power fields (HPFs). Tumor budding invasion margin was read and recorded under 10 HPFs. Tumor budding was quantified two ways: (1) the maximum number of tumor budding per HPF among the 10 HPFs (maximum budding/1 HPF) and (2) the total number of tumor budding of 10 HPFs (total budding/10 HPFs). In line with methodologies from existing literature, the smallest invasive tumor nest was classified into cell nest (composed of >1 tumor cell) and single cell invasion (**Figure 2C**). The size of the smallest tumor nest was assessed two ways: (1) the tumor nests in entire tumor area and (2) the tumor nests infiltrating the tumor edge.

The primary tumor’s 5-μm thick, hematoxylin and eosin (HE)-stained slides underwent microscopic analysis. The proportion of stromal tissue within the most invasive tumor region was systematically graded in increments of 10%, utilizing a 10× objective. This grading necessitated the visibility of tumor cells at all four peripheries of the field being viewed. Based on this analysis, the tumor-stroma ratio (TSR) was categorized as either stroma-poor (with less than 50% stroma content) or stroma-rich (with stroma content equal to or exceeding 50%).

Evaluation of nuclear features was performed at ×100 magnification to identify the regions exhibiting the most pronounced abnormal nuclear characteristics. The initial step involved scanning all tumor slides to select at least three HPFs showcasing the largest nuclear diameters. The criteria for nuclear size were aligned with LSCC, wherein nuclei considered ‘large’ exceeded the size of four small lymphocytes. Mitotic count was evaluated in the zones displaying the highest mitotic activity and was calculated per 10 HPFs (2.37 mm^2^ area) (20).

### Statistical analysis

The analysis of the relationships between variables was conducted employing the χ^2^ test for those that are categorical, and the two-sample t-test was utilized for variables that are continuous. This study delved into two critical endpoints: OS and DFS. The calculation of both OS and DFS was executed through the Kaplan-Meier method. The examination of the associations between pathological factors and the survival rates was thoroughly conducted using the log-rank test.

OS was defined as the period extending from the surgical intervention to either the unfortunate event of death or the last recorded follow-up. DFS was specifically defined concerning the time until disease progression. Multivariate analyses were expertly conducted using the Cox proportional hazards regression model with Forward LR method. For each of the two outcomes, the multivariate models incorporated all significant factors that were identified through rigorous univariate analyses. Any associations between pathological factors were checked, and if there were any strong associations discovered, only one factor was included into the model for analysis.

All statistical tests conducted in this study were two-sided, adhering to a 5% significance level as the threshold for determining statistical significance. Statistical analyses were performed using R (version 3.0.1; R Development Core Team) with the “maxstat,” “survival,” and “cmprsk” packages.

## Results

### Patient clinical characteristics

The study included 326 patients with a median age of 60 years (range, 37–76 years). The majority was male (87.7%). Pathological stages II and III were the most common, accounting for 54% and 40.2% of patients, respectively. Regarding the N stage, 39.9% of patients were classified as N0, while 60.1% had positive lymph node metastasis (N+). Nearly half (50.6%) exhibited moderate differentiation, with well and poor differentiation at 21.5% and 27.9%, respectively. The tumor lesion located in the middle thoracic esophagus account for 57.7% of cases. Vascular and perineural invasions were reported in 43.6% and 51.2% of patients. Throughout the study, 56.4% of patients (n = 184) experienced a recurrence, and 50.6% (n = 165) died from related and unrelated causes. The median follow-up was 42.2 months (range, 0.11–110.3 months), with a 5-year overall survival (OS) rate of 47%. The patient characteristics and their associations with prognosis were summarized in **Table 1**.

**Table 1:** Patient characteristics and their associations with prognosis.

	Overall Survival				Disease Free Survival			
	*N*	(%)	5-Year OS	*P*	*N*	(%)	5-Year DFS	*P*
**Sex**				0.07				0.27
Male	286	87.7	44.7		286	87.7	34.1	
Female	40	12.3	58.2		40	12.3	47.2	
**Age**				0.99				0.45
≤60	180	55.2	49.7		180	55.2	37.1	
>60	146	44.8	44.6		146	44.8	33.8	
T stage								
T1-2	138	42.3	52.7	0.18	138	42.3	39.5	0.53
T3-4	188	57.7	43.0		188	57.7	33.1	
**N stage**				<0.01				<0.01
N0	130	39.9	68.1		130	39.9	57.4	
N(+)	196	60.1	32.4		196	60.1	19.1	
**Differentiation**				<0.01				0.001
High	70	21.5	67.6		70	21.5	54.3	
Middle	165	50.6	45.2		165	50.6	34.3	
Low	91	27.9	33		91	27.9	25.8	
Site(7th)				0.69				0.94
Upper thoracic	68	20.9	44.3		68	20.9	33.7	
Middle thoracic	188	57.7	45.2		188	57.7	35.3	
Low thoracic	70	21.5	54.6		70	21.5	38.8	
**TNM stage**				<0.01				<0.01
I	19	5.8	79.4		19	5.8	75.2	
II	176	54	59.2		176	54	48.3	
III	131	40.2	26.4		131	40.2	12.9	
**Vascular invasion**				0.001				0.002
Yes	142	43.7	36.9		142	43.7	28.1	
No	183	56.3	55.9		183	56.3	42	
**Perineural invasion**				0.012				0.05
Yes	167	51.5	40.5		167	51.5	31.1	
No	157	48.5	52.9		157	48.5	40.4	

### Associations between histological and nuclear factors and differentiation

Pathological features’ correlations were analyzed across different differentiation groups. In the well-differentiated group, 94.3% exhibited a keratinizing pattern, compared to 52.2% in the poorly differentiated group. Nonkeratinizing patterns were significantly more prevalent in poorly differentiated tumors (45.7%) than well-differentiated ones (5.7%). Basaloid patterns were rare, found in only three patients, all within the moderately differentiated group.

The mean tumor budding invasion margin was significantly higher in the poorly differentiated group than in the well-differentiated group (36.3% vs. 25.3%, P<0.05, **Table 2**). Similarly, the number of tumor budding cells over 10 HPFs was more significant in poorly differentiated tumors (13 vs. 8.8, P<0.05). Based on tumor budding grading used in colon cancer (G1: 0-4 cells, G2: 5-9 cells, G3: ≥10 cells, **Supplementary Figure S1**), the proportion of G3 in poorly differentiated tumors was nearly double that in well-differentiated tumors (55.6% vs. 31.8%) under 10 HPFs (P<0.05, **Table 2**). The G1/G2/G3 grades distribution across well, moderate, and poorly differentiated groups showed no clear pattern, suggesting that ESCC tumor budding grading may differ from colon cancer.

**Table 2:** Pathologic features under differentiation landscape.

Differentiation										
	Well			Middle			Poor			
	*N*	%	Average	*N*	%	Average	*N*	%	Average	*P*
Subtype										
Keratinizing	66	94.3%		129	78.7%		48	52.2%		**<0.05**
Nonkeratinizing	4	5.7%		34	20.7%		42	45.7%		
Basaloid pattern	0	0.0%		1	0.6%		2	2.2%		
**Tumor budding invasion margin(%)**			25.3			30.7			36.3	**<0.05**
**Tumor budding (1HPF)**			3.0			3.1			3.8	**<0.05**
G1	52	78.8%		135	81.8%		59	64.8%		**<0.05**
G2	13	19.7%		30	18.0%		30	33.0%		
G3	1	1.5%		0	0.0%		2	2.2%		
**Tumor budding (10HPFs)**			8.8			10.3			13.0	**<0.05**
G1	26	39.4%		47	28.8%		21	23.3%		**<0.05**
G2	19	28.8%		54	33.1%		19	21.1%		
G3	21	31.8%		62	38.1%		50	55.6%		
Tumor nest-edge										
single cell present	42	60.9%		123	74.5%		66	72.5%		NS
nest cells	27	39.1%		42	25.5%		25	27.5%		
Tumor nest-entire										
single cell present	48	69.6%		140	84.8%		76	83.5%		**<0.05**
nest cells	21	30.4%		25	15.2%		15	16.5%		
Nuclear diameter										
≤4 Lymc.	29	41.4%		160	97.0%		85	93.4%		**<0.001**
≥5Lymc.	41	58.6%		5	3.0%		6	6.6%		
**Mitotic count**			25.0			14.9			17.6	**<0.05**
<15	28	40.0%		97	59.9%		45	50.6%		**<0.05**
≥15	42	60.0%		65	40.1%		44	49.4%		
**Tumor-Stroma Ratio(%)**			24.1			25.5			23.6	NS
<50%	60	85.7%		146	88.5%		77	84.6%		NS
≥50%	10	14.3%		19	11.5%		14	15.4%		
Invasion depth										
submucosa	13	18.6%		17	10.4%		2	2.2%		**<0.05**
muscularis propria	20	28.6%		90	54.9%		54	60.0%		
adventitia	37	52.9%		57	34.8%		34	37.8%		
Perineural invasion										
No	32	45.7%		87	53.4%		39	42.4%		NS
Yes	38	54.3%		76	46.6%		53	57.6%		
Lymphovascular invasion										
No	51	72.9%		92	56.1%		41	44.6%		**<0.05**
Yes	19	27.1%		72	43.9%		51	55.4%		

Bold values mean P<0.05.

Single-cell invasion and tumor nest presence were assessed in ITF areas, with single-cell invasion rates of 60.9%, 74.5%, and 72.5% in well, moderate, and poorly differentiated groups, respectively. Tumor nest presence were 39.1%, 25.5%, and 27.5%, respectively, with no significant difference between groups (P=0.10, **Table 2**). Nuclear diameter varied significantly across differentiation groups, with small diameters (≤4 lymphocytes, **Supplementary Figure S2**) observed in 41.4%, 97%, and 93.4% of well, moderate, and poorly differentiated tumors, respectively. Large diameter defined as ≥5 lymphocytes (**Figure 2D**) occur 58.6%, 3% and 6.6% in well, moderate and poorly differentiated groups, respectively (P<0.001, **Table 2**).

Mitotic counts varied significantly among differentiation groups (P<0.05), with mean counts of 25, 14.9, and 17.6 in well, moderate, and poorly differentiated groups, respectively. Using 15 as the cutoff value, based on criteria from LSCC, higher mitotic counts were more frequent in well-differentiated tumors (60%) than lower counts (40%). However, in middle differentiation group, more mitotic counts were reduced in comparison of less mitotic counts (40.1%vs. 59.9%). No difference of mitotic counts was observed in poor differentiation group (**Table 2**).

The mean stroma proportion in three groups was 24.1%, 25.5%, and 23.6%, respectively, showing no significant difference. According to previous research in LSCC, TSR was categorized as stroma poor (<50% stroma) or stroma rich (≥50% stroma). This analysis revealed no significant difference in TSR across differentiation groups in ESCC, with similar proportions of stroma poor or rich among well, moderate, and poorly differentiated groups (**Table 2**).

Significantly more submucosa invasion (T1 stage) was observed in the well-differentiated group compared to the poorly differentiated group (18.6% vs. 2.2%, P<0.05). Muscularis propria invasion (T2 stage) was more common in poorly differentiated tumors (60%) than in well-differentiated ones (28.6%). However, the incidence of adventitia invasion (T3 stage) did not significantly differ between groups. As a recurrence risk factor, perineural invasion rates were similar across all differentiation groups: 54.3% in well, 46.6% in moderate, and 57.6% in poorly differentiated tumors, indicating no significant difference. Lymphovascular invasion, a predictor of recurrence and metastasis, was nearly double in the poorly differentiated group compared to the well-differentiated group (55.4% vs. 27.1%, **Table 2**).

### Correlations between histological and nuclear factors and clinicopathological features

All the correlation analysis was summarized in **Supplementary Table S1**. Keratinizing subtypes were associated with more G1 tumor budding in the ITF, larger nuclear diameters, and N0 stage (P<0.05). Nonkeratinizing subtypes correlated with more G2/G3 tumor budding, smaller nuclear diameters, and positive lymph node status (N+). G1 tumor budding was linked to poor stroma and the absence of lymphovascular invasion (P<0.05). Single-cell invasion in ITF correlated with smaller nuclear diameters, deeper invasion, and more lymphovascular invasion (P<0.05). Smaller nuclear diameters were associated with fewer mitotic counts, deeper invasion, and increased lymphovascular invasion (P<0.05).

No significant correlations were found between mitotic counts and TSR, invasion depth, perineural invasion, lymphovascular invasion, T stage, or N stage. Fewer mitotic counts were only correlated with smaller nuclear diameters. Stroma’s poor status was associated with tumor budding, superficial invasion, absence of perineural invasion, early T stage (T1 + 2 vs. T3 + 4), and more lymph node metastasis. TSR was the only histological or nuclear factor correlating with the T and N stage.

In our current analysis, no significant correlation of single cell invasion in ITF and T stage and N stage (T1-2 vs. T3-4, P=0.11 and N(0) VS. N (+), P=0.05, respectively, **Supplementary Table S1**), suggesting a lack of significant correlation between single-cell invasion and TNM staging.

### Histological and nuclear features and their associations with OS and DFS

**Figure 3:**
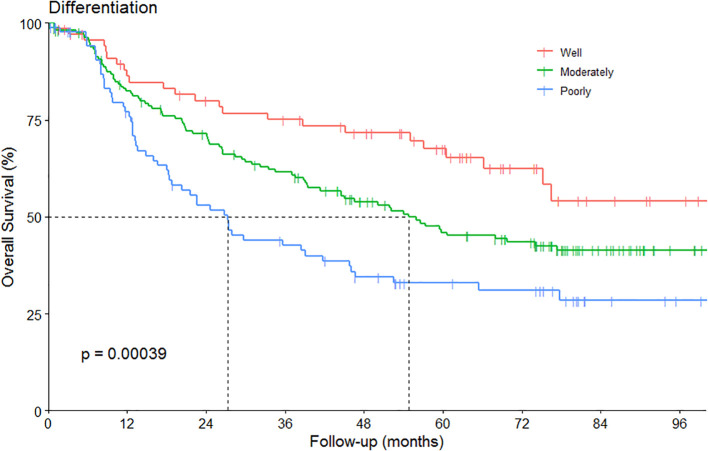
The overall survival (OS) according to the differentiation.

**Figure 4:**
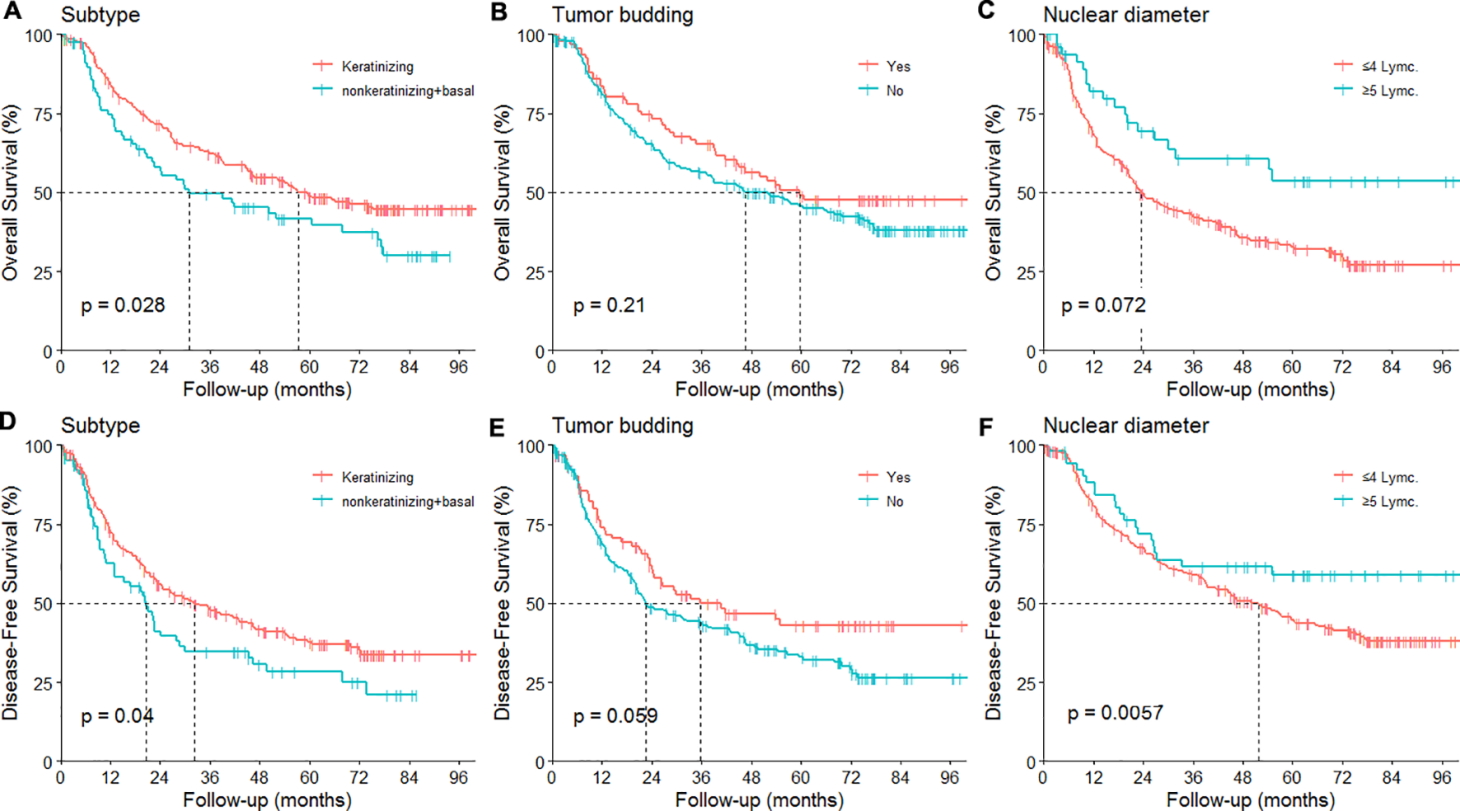
Prognosis based on pathological features. **(A–D)** overall survival (OS) and disease free survival (DFS) under different subtype; **(B–E)** OS and DFS under different tumor budding; **(D–F)** OS and DFS under different nuclear diameter.

The OS curve based on differentiation was showed in **Figure 3**. The associations between the histological, nuclear features and prognosis were summarized in **Table 3**. A notable result was the improved OS and DFS associated with the presence of keratinization (P = 0.03 and 0.04, respectively, **Figures 4A, D**). Regarding the invasion margin, with a mean value of 31% and using a cutoff of 40%, no significant differences in 5-year OS and DFS were observed. Similarly, no significant difference in 5-year OS and DFS was noted when using 5 buds/HPF as the cutoff. However, with a cutoff of 5 buds per 10 HPFs, patients with high-grade tumor budding (≥5 buds/10 HPFs) exhibited significantly worse 5-year DFS compared to those with low-grade budding (<5 buds/10 HPFs; P = 0.05, **Figures 4B, E**). A beneficial trend toward better 5-year OS was observed, although it was not statistically significant.

**Table 3:** Pathologic features and their associations with prognosis.

	OS				DFS			
	*N*	%	5-year OS	*P*	*N*	%	5-year DFS	*P*
Subtype								
Keratinizing	242	74.5%	48.7%	**0.03**	241	74.4%	37.8%	**0.04**
Nonkeratinizing/Basaloid	83	25.5%	39.6%		83	25.6%	28.2%	
**Tumor budding invasion margin(%)**				0.49				0.73
<40	199	61.0%	48.6%		198	60.9%	39.2%	
≥40	127	39.0%	44.1%		127	39.1%	29.8%	
**Tumor budding (1HPF)**				0.60				0.23
<5	246	76.4%	47.5%		190	59.2%	39.1%	
≥5	76	23.6%	43.3%		131	40.8%	29.6%	
**Tumor budding (10HPFs)**				0.21				**0.05**
<5	94	29.5%	49.1%		93	29.2%	43.0%	
≥5	225	70.5%	45.7%		225	70.8%	33.0%	
Tumor nest-edge								
single cell present	231	71.1%	46.3%	0.99	231	71.3%	35.3%	0.81
nest cells	94	28.9%	48.0%		93	28.7%	35.7%	
Nuclear diameter								
≤4 Lymc.	274	84.0%	44.6%	**0.07**	274	84.3%	32.7%	**0.006**
≥5 Lymc.	52	16.0%	59.0%		51	15.7%	53.6%	
**Mitotic count**				0.54				0.82
≤15	173	53.9%	45.7%		173	54.1%	35.7	
>16	148	46.1%	48.8%		147	45.9%	35.6	
Tumor-Stroma Ratio(%)								
<50%	283	86.8%	46.4%	0.42	282	86.8%	35.1%	0.23
≥50%	43	13.2%	50.6%		43	13.2%	39.3%	
Invasion depth								
submucosa/muscularis propria	196	60.5%	48.7%	0.54	195	60.4%	35.4%	0.65
adventitia	128	39.5%	44.4%		128	39.6%	36.3%	
Perineural invasion								
No	157	48.5%	52.9%	**0.01**	157	48.6%	40.4%	**0.05**
Yes	167	51.5%	41.3%		166	51.4%	29.9%	
Lymphovascular invasion								
No	183	56.3%	55.9%	**0.001**	182	56.2%	42.0%	**0.002**
Yes	142	43.7%	36.9%		142	43.8%	28.1%	

OS, overall survival; DFS, disease free survival. Bold values mean P<0.05.

The analysis also revealed that the presence of single-cell invasion in the ITF did not significantly impact 5-year OS and DFS (P > 0.05, **Table 3**). Nuclear diameter, categorized into small (≤4 lymphocytes) and large (>5 lymphocytes), showed that patients with larger nuclear diameters had better OS and DFS than those with smaller diameters. Specifically, the 5-year OS rate for patients with larger nuclear diameters was 53.6%, compared to 32.7% for those with smaller diameters (P = 0.006, **Figures 4C, F**). Larger nuclear diameters were also associated with the absence of lymphovascular invasion.

The mean value of mitotic count and TSR in ESCC are 17.8 and 24.7%. No significant difference in 5-year OS and DFS was observed based on mitotic counts and the TSR, using cutoff values 15 for mitotic counts and 50% for TSR according to the criteria in the LSCC (P>0.05). The presence of lymphovascular invasion and perineural invasion were associated with a worse OS and DFS in univariate analysis (P<0.05, **Table 3**).

### Multivariate analysis of OS and DFS

In the univariate analysis, factors such as histological subtype, nuclear diameter, perineural invasion, and lymphovascular invasion were strongly associated with OS (P < 0.05). The same factors and tumor budding were associated with DFS (P < 0.05). These risk factors were included in the multivariate analysis for OS and DFS, which identified the nuclear diameter as independent factors affecting both OS and DFS. The study did not find independent prognostic significance for tumor budding or single cell invasion. The analysis results were summarized in the **Table 4**.

**Table 4:** Multivariate analysis of overall survival and disease free survival.

		OS			DFS		
		HR	95%CI	*P*	HR	95%CI	*P*
**Subtype**	Keratinizing vs. Nonkeratinizing	1.23	0.87-1.76	0.242	1.14	0.82-1.60	0.446
**Tumor budding**	<5/HPF vs. ≥5/HPF	0.96	0.65-1.41	0.823	0.88	0.61-1.27	0.476
**Tumor budding**	<5/10HPF vs. ≥5/10HPF	1.19	0.82-1.74	0.368	1.36	0.94-1.95	0.104
**Nuclear diameter**	≤4 Lymc. vs. ≥5 Lymc.	0.59	0.36-0.96	**0.035**	0.48	0.28-0.80	**0.005**
**Perineural invasion**	No vs. Yes	1.29	0.92-1.80	0.14	1.16	0.85-1.59	0.358
**Lymphovascular invasion**	No vs. Yes	1.10	0.78-1.55	0.585	1.04	0.75-1.43	0.823
**Pathological stage**	II vs. I	0.23	0.09-0.63	**0.004**	0.14	0.05-0.45	**0.001**
	III vs. I	0.39	0.28-0.53	**<0.001**	0.40	0.30-0.54	**<0.001**

HPF, High power field; Lymc., lymphocytes. Bold values mean P<0.05.

## Discussion

We identified pathological features across differentiation landscapes in ESCC. Characteristics such as the keratinizing subtype, smaller tumor invasion margins, more G1 tumor budding, absence of single cell invasion, larger nuclear diameters, higher mitotic counts, shallower infiltration, and less lymphovascular invasion were more likely to occur in well-differentiated ESCC. But poorly differentiated ESCC exhibited conversely.

Regarding tumor budding invasion margin, previous few studies have reported in ESCC. In our study, the invasion margins for well, moderate, and poorly differentiated ESCC were 25.3%, 30.7%, and 36.3%, respectively, providing valuable data for pathologists determining the degree of differentiation. A universally accepted cutoff for tumor budding in ESCC has yet to be established. We found mean values of tumor budding per HPF in well, moderate, and poorly differentiated tumors to be 3.0, 3.1, and 3.8, respectively, and the values under 10 HPFs to be 8.8, 10.3, and 13, respectively.

Referring to tumor budding grades in colon cancer, our analysis showed that more G3 tumor budding per 10 HPFs occurred in poorly differentiated tumors (55.6% vs. 31.8% in well-differentiated tumors). We can see that the poor differentiation is associated with more giant tumor budding invasion margins and more tumor budding cells (under 10 HPFs), nearly double that in well-differentiated tumors. In prognostic analysis, fewer tumor buds under 10 HPFs correlated with better DFS. Previous research has identified tumor budding as a prognostic factor in endometrioid endometrial cancer, colon cancer, and gastric cancer (21–23). However, the cutoff value varies across studies, even within the same pathological tumor type for LSCC (1, 24). Kadota et al. defined low (0–9 buds/10 MPF) and high tumor budding (≥10 buds/10 MPF), while Weichert et al. categorized budding into low (0/10 MPF), intermediate (1–14 buds/10 MPF), and high (≥15 buds/10 MPF) groups.

Regarding the classification of MTN, they were divided into four categories: large nest (≥15 tumor cells), intermediate nest (5–14 tumor cells), small nest (2–4 tumor cells), and single-cell invasion (1, 24). Our analysis focused on MTN at the ITF. It revealed that single-cell invasion was more common than tumor nests in ESCC (mean value 70.9% vs. 29.1%). Previous research has demonstrated that a markedly lower 5-year DFS for patients exhibiting single-cell invasion compared to those presenting with small cellular clusters in lung cancer (1, 25).

Tumors that exhibit single cell invasion are identified as exceedingly malignant that is different from less malignant invasive cancers, which may present with either large or small tumor nest components. Our findings indicated that single-cell invasion is more prevalent in poorly and moderately differentiated tumors than in well-differentiated ones (72.5-74.8% vs. 60.9%), aligning with observations in LSCC. However, the employing small tumor clusters of ≤15 tumor cells as a histologic risk model in head and neck squamous cell carcinomas did not serve as a prognostic indicator for ESCC in our study (7).The difference of OS and DFS either based on the single cell invasion or the tumor nest presence were both not significant. This is the first time to identify the clinical significance of the single cell invasion and tumor nest in the ESCC. Of course, the value of single cell invasion in the ESCC warrants further validation. Notably, Kadota et al. identified single-cell invasion as an independent prognostic factor. However, it did not hold the same significance in stage I patients, suggesting a correlation between single-cell invasion and tumor stage (1). In the current analysis, the single cell invasion in the ITF did not correlate with T stage categorical variable (T1-2 vs. T3-4) and N stage. In other words, single cell invasion did not show significant correlation with TNM stage. Maybe this is the reason that single cell invasion did not play as the independent prognostic factor. Consistent with another research, single-cell invasion was also associated with lymphovascular invasion in our cohort. The incidence of single-cell invasion was significantly higher in specimens with lymphovascular invasion than those without (92.1% vs. 78.3%), highlighting its potential role in assessing tumor aggressiveness and metastatic potential (2).

Previous studies have typically used a 50% cutoff value to differentiate stroma-poor and stroma-rich tumors. In Wang’s comprehensive study, it was meticulously observed that the three-year OS and DFS rates exhibited a marked increase in the stroma-poor cohort (26). In our analysis, no significant correlation between TSR and prognosis might be attributed to differences in sample sizes between studies (95 in Wang Kai’s study versus 326 in ours). Most specimens in our cohort were stroma-poor (86.8%) compared to stroma-rich (13.2%), based on the 50% cutoff. The proportion difference between stroma-poor and stroma-rich in Wang’s research was 68.4% to 31.6%, which was not as pronounced in our analysis. TSR, a crucial component of the tumor microenvironment, may facilitate tumor cell proliferation and contribute to tumor progression (27, 28).

Regarding nuclear features, such as nuclear diameter and mitotic count, established grading systems exist for kidney and breast cancers (29, 30). In the current analysis, we found more large nuclei in well differentiation and more small nuclei in poor differentiation. The small nuclear diameter was significantly associated with lymphovascular invasion. And patients with small nuclear diameter had worse DFS and OS. The results was same with lung adenocarcinoma but different from the LSCC where large nuclei was independently associated with a worse OS; but this was done after stratifying by pathologic stage (1). Our results showed that nuclear diameter may predict prognosis in multivariate analysis. In further analysis, the nuclear diameter will be verified in large cohort of pathologic specimen.

For mitotic count, using a cutoff of 15/HPF, we observed that a higher mitotic count was more likely in well-differentiated tumors and associated with larger nuclear diameters. Yet, the mitotic count did not serve as a prognostic factor for OS and DFS in ESCC, echoing findings in LSCC (1, 2). In contrast, lung adenocarcinoma studies have shown that the recurrence-free probability of patients with high mitotic count (≥5/10 HPF) was the lowest followed by intermediate (2-4/10 HPF) and low (0-1/10 HPF, P<0.001) (20). We can see that the cut-point is different in lung squamous cell carcinoma and adenocarcinoma (15 vs.5). The mean value of mitotic counts in the ESCC is 17.8. If we choose 5 as the cut-point, the sample size in each group varied significantly thus analyzed deviation will be significant.

In the multivariate analysis of OS and DFS, except for TNM stage, only nuclear diameter was identified as independent prognostic factors. Pathological characteristics such as pathologic subtype, and tumor budding despite showing significant prognostic value in univariate analyses, did not emerge as prognostic factors in the multivariate analysis for ESCC. Although in the LSCC, total tumor budding (HR = 1.04), single cell invasion in the entire tumor (HR = 1.47), and single cell invasion in the tumor edge (HR = 1.49) were found to be independent prognostic factors for worse prognosis. However, tumor budding and single cell invasion were significantly associated with other prognostic factors (such as TNM stage and lymphovascular invasion), and the HRs hovering close to 1 suggest limited clinical utility (1). This highlights an urgent call for further identification of tumor budding and single-cell invasion in clinical prognostication.

There are some limitations in the present study. Our investigation is a retrospective study. There was no opportunity to evaluate the basaloid squamous cell carcinoma because of the rare incidence. The single cell invasion in ESCC did not play as the prognostic factor like in the grading systems for the LSCC and other cancers. The point has been to be validated in different, larger cohorts in future.

In conclusion, our research delineated the pathological features across different differentiation landscapes in ESCC. Well-differentiated tumors were likelier to exhibit a keratinizing subtype, more G1 tumor budding, larger nuclear diameter, higher mitotic counts, superficial tumor infiltration, and less lymphovascular invasion. Conversely, poor differentiation was characterized by more G2/3 tumor budding. The average tumor budding invasion margin was 25.3% in well-differentiated tumors, compared to 36.3% in poorly differentiated ones. These findings can aid pathologists in determining tumor differentiation. In the prognosis multivariate analysis, nuclear diameter based on the cut-off value with 4 lymphocytes proved to be more predictive of OS and DFS than other pathological features, including subtype, tumor budding, single-cell invasion, and tumor stroma.

## Data Availability

The raw data supporting the conclusions of this article will be made available by the authors, without undue reservation.

## Supplementary material

Supplementary Figure 1 Tumor budding grade according to the colon cancer criteria. **(A)** G1; **(B)** G2, **(C)** G3.

Supplementary Figure 2 Nuclear diameter. The circle represents four lymphocytes. The red arrows represent small nuclear diameter. The black arrows represent large nuclear diameter.

Supplementary Table 1 Correlations between pathologic features.
