# Supplementary figures and images for: Pathological features of the differentiation landscape in esophageal squamous cell cancer and their correlations with prognosis
Source: Front Oncol. 2024 Dec 6;14:1442212. doi: 10.3389/fonc.2024.1442212 (PMC11659131; doi:10.3389/fonc.2024.1442212)

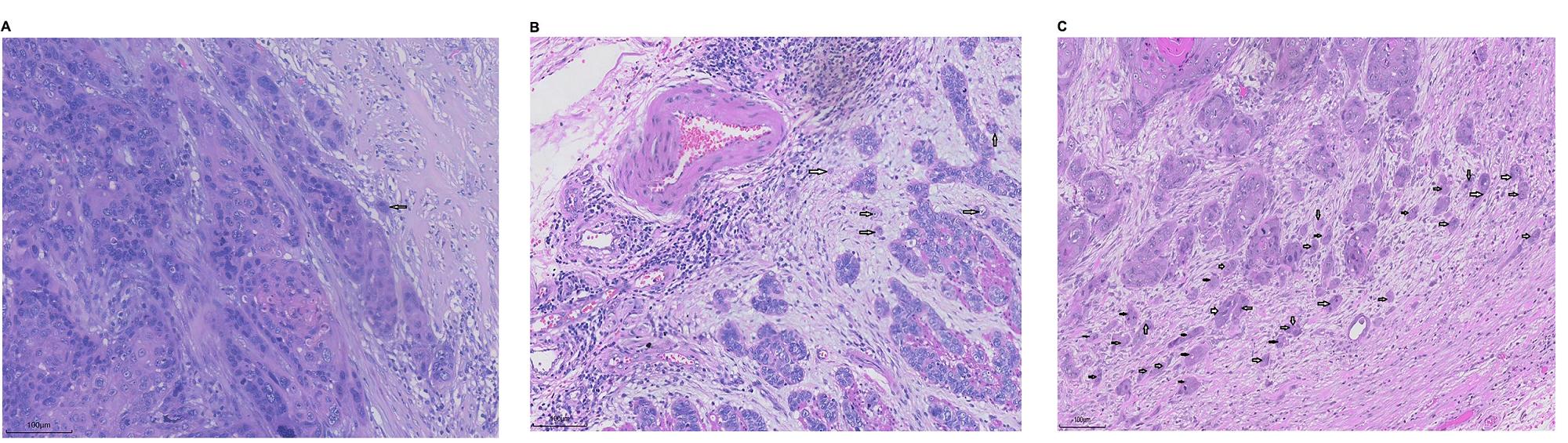

Supplement: Supplementary Figure 1 — Tumor budding grade according to the colon cancer criteria. (A) G1; (B) G2, (C) G3. [file Image1.tif]

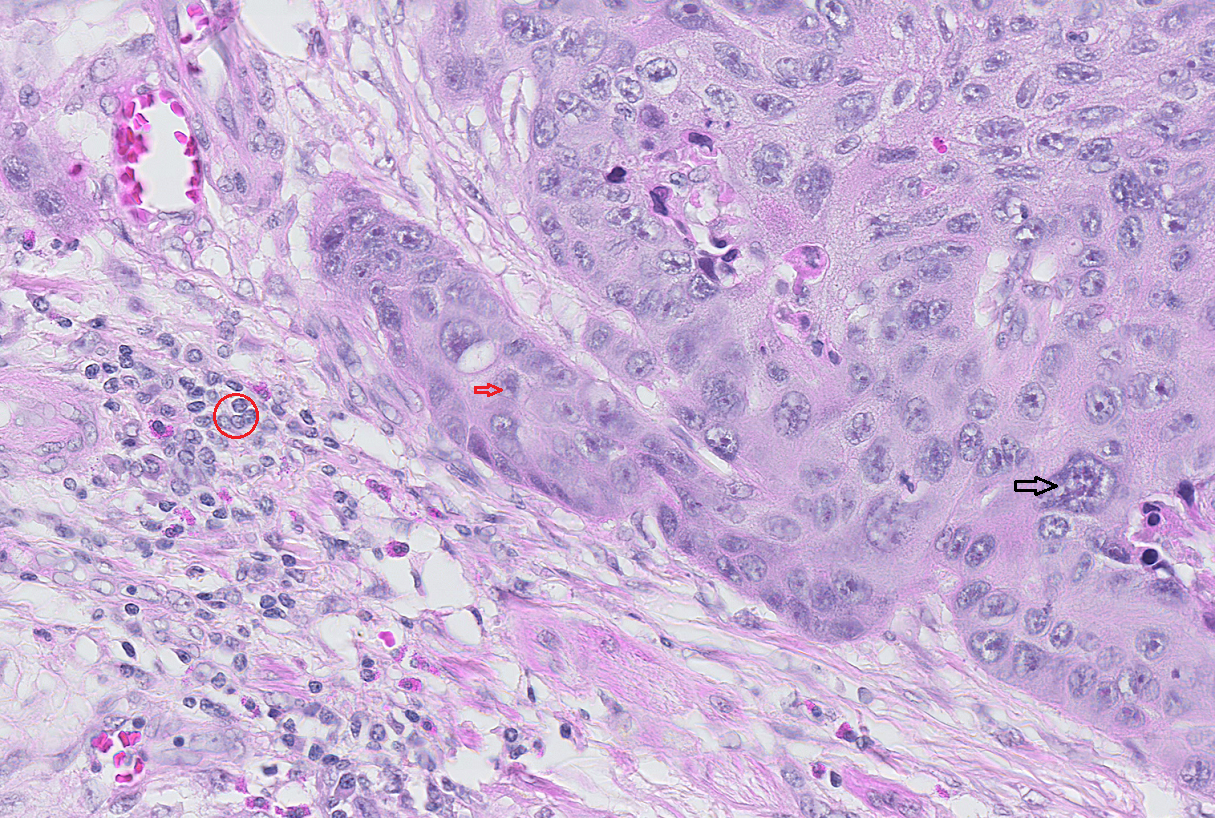

Supplement: Supplementary Figure 2 — Nuclear diameter. The circle represents four lymphocytes. The red arrows represent small nuclear diameter. The black arrows represent large nuclear diameter. [file Image2.png]
